# *Leishmania donovani* infection induce differential miRNA expression in CD4+ T cells

**DOI:** 10.1038/s41598-020-60435-2

**Published:** 2020-02-26

**Authors:** Vinod Kumar, Sushmita Das, Ajay Kumar, Neeraj Tiwari, Ashish Kumar, Kumar Abhishek, Abhishek Mandal, Manjay Kumar, Taj Shafi, Tanvir Bamra, Rakesh Kumar Singh, Saravanan Vijayakumar, Abhik Sen, Pradeep Das

**Affiliations:** 1 Department of Molecular Biology, Rajendra Memorial Research Institute of Medical Sciences, Agamkuan, Patna, Bihar India; 2 Department of Microbiology, All India Institute of Medical Sciences, Phulwarisharif, Patna, Bihar India; 3 Department of Biochemistry, Rajendra Memorial Research Institute of Medical Sciences, Agamkuan, Patna, Bihar India; 4 Department of Bioinformatics, Rajendra Memorial Research Institute of Medical Sciences, Agamkuan, Patna, Bihar India; 5 Department of Biochemistry, Institute of Science, Banaras Hindu University, Varanasi, India

**Keywords:** Diseases, Parasitic infection

## Abstract

Visceral leishmaniasis is characterized by mixed production of Th1/2 cytokines and the disease is established by an enhanced level of Th2 cytokine. CD4+ T cells are main cell type which produces Th1/2 cytokine in the host upon *Leishmania* infection. However, the regulatory mechanism for Th1/2 production is not well understood. In this study, we co-cultured mice CD4+ T cells with *Leishmania donovani* infected and uninfected macrophage for the identification of dysregulated miRNAs in CD4+ T cells by next-generation sequencing. Here, we identified 604 and 613 known miRNAs in CD4+ T cells in control and infected samples respectively and a total of only 503 miRNAs were common in both groups. The expression analysis revealed that 112 miRNAs were up and 96 were down-regulated in infected groups, compared to uninfected control. Nineteen up-regulated and 17 down-regulated miRNAs were statistically significant (p < 0.05), which were validated by qPCR. Further, using *insilco* approach, we identified the gene targets of significant miRNAs on the basis of CD4+ T cell biology. Eleven up-regulated miRNAs and 9 down-regulated miRNAs were associated with the cellular immune responses and Th1/2 dichotomy upon *Leishmania donovani* infection. The up-regulated miRNAs targeted transcription factors that promote differentiation of CD4+ T cells towards Th1 phenotype. While down-regulated miRNAs targeted the transcription factors that facilitate differentiation of CD4+ T cells towards Th2 populations. The GO and pathway enrichment analysis also showed that the identified miRNAs target the pathway and genes related to CD4+ T cell biology which plays important role in *Leishmania donovani* infection.

## Introduction

Leishmaniasis has been included among thirteen neglected tropical parasitic diseases by world health organization (WHO), which is caused by intracellular protozoan parasites of the genus *Leishmania*. The parasite is transmitted by the bite of infected sand fly^1^ to the human host. The disease pathogenesis is categorized into three forms, i.e. visceral, mucocutaneous (MCL) and cutaneous leishmaniasis (CL) based on the clinical presentations. About 7, 00,000 to 1.2 million new cases are estimated to occur annually that eventually results in death of about 20,000 to 40,000 per year^1,2^.

Visceral leishmaniasis (VL), also known as kala-azar is more prevalent in the Indian continent, East Africa and Brazil. Visceral infections are almost fatal if left untreated^1,3^. An estimated 50,000 to 90,000 new cases of VL are annually reported from mainly seven countries i.e. Brazil, Ethiopia, India, Kenya, South Sudan, Somalia and Sudan^3^. VL symptoms include fever, anemia, weight loss and enlargement of the liver and spleen.

Immune cells such as B & T cells, neutrophils and macrophage are the main players of immune responses in leishmaniasis^4^. Among T cells, the CD4+ T cells are known to play a central role in regulating cellular immune responses during *Leishmania donovani* infection^5^. After *Leishmania* infection, naive CD4+ T cells differentiate into specific T cells type such as T regulatory (Treg), Th17, Th1 and Th2 depending upon immune response and expression of specific transcription factors^6^. Each T cell subtype has specific functions and secretes different cytokines that largely depend on host and parasite. The CD4+ T cells secrete interferon-gamma (IFN-γ), which activates the JAK-STAT pathway and transcription of STAT-1, STAT-4 and T-bet^7^. This pathway further induces IL-12 signaling and inducible nitric oxide synthase (iNOS) expressions which are required for killing the parasites^8^. On the other hand IL-4, IL-2, and IL-13 which are major anti-inflammatory cytokines secreted by CD4+ T cells, help in the survival of parasites inside the macrophage by activating STAT-3 which inturn induces the secretion of anti-inflammatory cytokines by macrophages^9,10^.

MicroRNAs (miRNAs) are single-stranded molecules having length of 19 to 25 nucleotides. They have been found to play a significant role in various human disease pathogeneses^11^. MicroRNAs pairs with untranslated regions (UTR) of the target genes and inhibit their functions. MicroRNAs regulate various functions of cells like apoptosis, stress response, immune response, differentiation and proliferation^12,13^. A single miRNA can target more than hundred to thousand genes and are also know to regulate DNA transcription, methylation, and histone acetylation as well^14^.

The role of miRNA in proliferation, differentiation, and development of CD4+ T cells during parasitic diseases has already been reported^15–17^. Studies suggest that miR-155, miR-181c, miR-9, and miR-31 play significant role in T cell activation by regulating IL-2 signaling pathway. Few miRNAs have been also reported to be associated with T-cell differentiation. miR-132/212 cluster are known to induce Th17 cell differentiation and its deficiency lowers the frequency of Th1 and Th17 cells in an experimental autoimmune encephalitis development^18^. Further, increased expression of miR-26a, has been shown to regulate the population of Treg cells, which eventually controls Th1 and Th17 differentiation^19^. In case of infectious diseases, pathogenesis caused by *Plasmodium spp*, *Leishmania major*, *Helicobacter pylori* and human immunodeficiency viruses, miRNAs have been reported to play important roles as immune regulators of host immunity^20–23^. However, in *Leishmania donovani* infection, the role of miRNAs in the differentiation and development of naive CD4+ T cells is not reported to date.

CD4+ T cells play a central role in immune regulation by secreting Th1/2 cytokines during *Leishmania donovani* infection. The effector properties of CD4+ T cells have been studied, but how these cells are regulated at the transcription level to differentiate into Th1 or Th2 phenotypes is not well understood. We hypothesized that miRNAs may act as a key factor in regulating the differentiation of naive CD4+ T cells into Th1/2 by regulating transcription factors. In the present study, we identified the miRNAs expressed in CD4+ T cells using next-generation sequencing during *Leishmania donovani* infection. This is the first study that explains the miRNA regulation of CD4+ T cell-mediated cellular immune response during *Leishmania donovani* pathogenesis.

## Material and Methods

### Ethics statement

All experiments in this study were performed following the ethics regulation of “Institutional Animal Ethical Committee” of the Rajendra Memorial Research Institute of Medical Sciences (Patna, India). Prior to the start of the experiments, an approval for protocol was taken from the “Institutional Animal Ethical Committee” of the Rajendra Memorial Research Institute of Medical Sciences Patna, India (Ref. No. 364/GO/R/S/2001/CPCSEA).

### Animals

Female mice (BALB/c, age between 6–8 weeks) were used in this study. The animals were provided with polypropylene cages and bedding material, wheat straw and optimum temperature of around 20–30 °C.

### *L. donovani* culture

A clone line strain AG83 of *L. donovani* was used for this study. The motile promastigotes were cultured in complete M199 medium (pH 7.2; Gibco, United States), containing 10% heat-inactivated fetal bovine serum (FBS; Gibco, United States), supplemented with penicillin (100 U/mL), gentamicin (20 mg/mL), streptomycin (100 mg/mL), 2 mM L-glutamine and sodium bicarbonate (3.7 gm/L) at 26 °C in a BOD incubator. Metacyclic promastigotes were used in all experiments, which were harvested from late log phage to the stationary stage using standard protocol.

### Isolation of macrophages (Mφ) from mice

Starch solution (4%) was administered into the peritoneal cavity of mice. After 48 h, macrophages were isolated using the standard protocol. Cells were washed with PBS and suspended in RPMI medium containing antibiotics and 10% FBS. Cells were incubated in a CO_2_ incubator with 5% CO_2_ atmosphere. After 12 h of incubation, the non-adherent cells were removed by washing and a fresh complete RPMI medium was added to the cells, plated in six-well culture plates (10^6^ cells/well).

### Macrophage infection with *L. donovani*

For macrophage infection, *Leishmania* parasites were incubated with macrophages (parasite/cell ratio is 10:1) for 6 h. After 6 h of interaction, the unbound parasites were removed by washing with incomplete RPMI media and infected macrophages were incubated in a CO_2_ incubator for 24 h as per our experimental conditions. The cells were fixed with formaldehyde and stained with May-Grünwald Giemsa and observed under a bright-field microscope at 100X magnification.

### Isolation of CD4+ T cells from BALB/c mice spleen

CD4+ T cells were isolated from the spleen of Balb/c mice using the standard protocol. Briefly, the mice were euthanized, and spleens were collected in a petri dish containing PBS (pH 7.2). Spleens were gently smashed with syringe plunger and cell suspension was prepared for isolation of CD4+ T-lymphocytes. Magnetic beads (Milteny Biotech Inc., USA) and anti-CD4-FITC antibodies (for positive selection) were used for isolation of CD4+ T cells following the protocol described elsewhere^24^. Briefly, the cell suspension was prepared at a concentration of 6 × 10^7^cells in 1 mL isolation buffer (1X PBS, pH 7.2, 0.5% BSA and 2 mM EDTA). Monoclonal antibody (2 µL/mL, anti-CD4-FITC) was added to the cell suspension and cells were incubated at room temperature (RT) for 15 min followed by addition of the magnetic microbeads (10 µL/mL) for 15 min at RT. Next, the cell suspension was loaded into MACS column (Milteny Biotech Inc.) and CD4+ T cells were isolated following manufacturer’s protocol. The CD4+ T cells, labeled with magnetic microbeads were retained on the column, whereas the unlabeled cells were passed through the column and the fraction was depleted of CD4+ T cells. After removal of the column from the magnetic field, the magnetic labeled CD4+T cells were eluted by washing the magnetic column with 15 mL of isolation buffer. The purity of CD4+ T-cells was measured by flow cytometry on a FACS ARIA II instrument (Becton Dickinson, USA). The results are shown as percentage of positive CD4+ T cells among the selected lymphocyte populations. CD4+ T cell viability was measured by Trypan blue dye exclusion method^25^. Briefly, isolated CD4+ cell suspension (100 µL) was taken in 1.5 ml tube and 100 µL Trypan Blue dye was added. After 5 minutes of incubation, the 10 µL of cell suspension was taken on haemocytometer with cover slip and observed under 40X light compound microscope (Olympus, Tokyo, Japan) and the unstained viable cells were counted.

### Macrophage- CD4+ T cells co-culture and RNA isolation

After purification, the live CD4+ T cells were co-cultured with *L. donovani* infected and non-infected macrophage for 36 h. The CD4+ T cells co-cultured with non-infected macrophages were considered as control. After co-culture, the CD4+ T cells were collected from culture supernatant and the viability of collected cells was analyzed by the Trypan blue dye exclusion method. Further, purity was checked prior to RNA isolation and miRNA sequencing. In the next step, RNA was isolated using standard protocol from both groups of CD4+ T cells by the Trizol method and highly pure RNA was processed for next-generation sequencing of miRNAs (Genotypic Technologies, India).

### Small RNA library preparation and next-generation sequencing

TrueSeq small RNA library prep kit (Illumina San Diego CA, USA) was used for the preparation of small RNA library and the protocol was followed as per manufacturer instruction. The procedure for small RNA library preparation included adapter ligation, reverse transcription, PCR amplification and pooled gel purification to generate a small RNA library. In the RNA sample, 3′ adapter was specifically modified to target miRNAs. After ligation of adapter to each end, the RNA was reverse transcribed to single-stranded cDNA and sequenced for preparation of small RNA library using an Illumina HiSeq 2000 platform (Illumina, San Diego, CA, USA). Further, using a DNA specific chip, library construct (1 μl) was loaded on Agilent Technologies 2100 Bioanalyzer for validation of small RNA library. Before the start of the sequencing, all the individual libraries were clustered together in a single lane on an Illumina HiSeq. 2000 platform using TrueSeq Cluster kit V3- cBot-HS (HiSeq) that generated 0–100 bp paired-end reads.

### Raw data generation and sequence filtration

The raw data of length 75 bp was generated on the Illumina platform and the data was received in FastQ format. Small RNA workbench V3.0_ALPHA1was used to trim 3′ adapter and performed length filtering (minimum length 16 bp and maximum 40 bp). The low quality and contaminated reads were removed on the following criteria to obtain final clean reads (i) Elimination of low quality reads (<q30), (ii) Elimination of reads without 3′ adapters, (iii) Elimination of reads without insert, (iv) Elimination of reads <16 bp and >40 bp, (v) Elimination of reads not matching to reference genome and (vi) Elimination of reads matching to other ncRNAs (r, t, sn, and snoRNAs). Conserved miRNAs were identified by a homology match approach against matured mouse miRNAs from miRBase and the novel miRNAs were recognized based on prediction of secondary structure.

### Identification of conserved miRNA

The raw data received in FastQ format was processed for identification of miRNAs. The high-quality clean reads were aligned to the *Mus musculus* genome using the software BOWTIE 1.1.1. The aligned reads were extracted and checked for ncRNA (rRNA, tRNA, snRNA and snoRNA) contamination. The unaligned reads to ncRNA database were used for known miRNA prediction. Repeated miRNAs were made unique by clustering approach using CD-HIT and read count profile was generated. Further, a homology search of these miRNAs was done against mouse mature miRNA sequences retrieved from miRbase-21 using ncbi-blast-2.2.30 with the threshold of e-3 and un-gapped alignment. The resulting outputs were miRNAs hits, with matching coordinates in the mouse genome and the precursor sequence. The miRNAs were matched with different variants of miRNAs such as different mature sequences that can arise from the same precursor, identified in miRBase.

### Identification of novel miRNA

Sequences not showing hits with known miRNAs were extracted and considered for novel miRNA prediction. The filtered sequences were further mapped to a genome of interest using BOWTIE software. Small RNAs reads between 16 and 25 nucleotides were considered for identification of novel miRNA. The sequences were mapped onto mouse genome with default parameters and miRNAs were identified by the Mireap_0.22b software. Further, the list of clusters was produced and analyzed to identify novel miRNA. The most abundant small RNA within a cluster was taken as the potential novel miRNA candidate. The flanking sequences around small RNA were taken from the genome and then folded using RNA fold software. Further, they were trimmed and the resulting secondary structures were analyzed to identify whether they form a miRNA hairpin. Additional checks were performed to make sure that there were no more than three consecutive mismatches, at least 1–25 nucleotide centered around the miRNA involved in base pairing, and the hairpin length is 75 nucleotide. The valid and most stable hairpin from each of the sequence windows was considered as the precursor miRNA candidate. After that, the precursor miRNA candidate was tested using software RNA fold (using a cutoff of 0.1).

### Differential expression (DE) analysis

Read count table for all the samples was generated and DE analysis was carried out using the DE-Seqtool. Variations in the read counts were normalized by the library normalization method. The read counts were normalized by dividing size factor calculated by DE-Seqtool. The normalized value of the given samples was used for DGE calculation and heatmap generation (log2fold of 1 was used as the cutoff). The normalized value of given miRNAs >1 were considered as “UP”, the miRNAs < −1 were considered as “DOWN” regulated and those between 1 and −1 were flagged as “NEUTRAL”. Heatmaps for differential expression of miRNAs were generated. The statistical significant (p < 0.05) dysregulated miRNAs expressed in both samples were taken for further study.

### Quantitative real time validation of dysregulated miRNAs

The Real-time PCR was performed to validate statistically significant dysregulated miRNAs. RNA was isolated from both groups of CD4+ T cells using TRI Reagent (Sigma Chemicals, United States) and the protocol provided by the manufacturer. Briefly, 1 × 10^6^ cells were taken from both groups and pelleted by centrifugation at 5,000 rpm. The pellet was washed with PBS (0.02 M, pH 7.2). Further, the cells were lyzed in 300 µL TRI reagent and 120 µL chloroform and centrifuged (10,000 rpm, 10 min). After centrifugation, the upper aqueous layer was pipette out and transferred into another centrifuge tube followed by addition of isopropanol (1:2 ratio). The mixture of aqueous layer and isopropanol was centrifuged again (10,000, 10 min) and RNA pellets were collected. Finally, RNA pellets were washed with 70% (v/v) ethanol (treated with RNAse-free DNase to avoid DNA contamination) and quantified by nanodrop spectrophotometer. In all step of centrifugation, the temperature was set at 4 °C.

For real time validation, 1 μg total RNA (kept equal for each amplification) was subjected to reverse transcription for cDNA synthesis using 20U M-MLV reverse transcriptase (Fermantas, Germany), 1X RT buffer, 20 mM dNTPs (New England Biolabs, USA), 20U RNasin (Fermentas, Germany), 0.1 M DTT with DEPC treated water and miRNA specific stem loop primers. The expression level of each miRNAs were quantified by Real Time PCR (ABI7500 Fast system, Applied Biosystem) as per manufacturer instructions using 5pmol/μl of specific primers with snoRNA 142 being taken as an endogenous control. Twenty microliter of real-time master mix contained 10 μl of SYBER green master mix (Applied Biosystem), 1 μl cDNA, 6 μl MilliQ water and 3 μl forward and reverse primers. PCR conditions were set with an initial incubation of 50 °C for 2 min, followed by denaturation at 95 °C for 10 min and 40 cycles at 95 °C for 15 s, 60 °C for 1 min, and 72 °C for 40 s. Results are expressed as target miRNA/reference miRNA, ratio of sample, and normalization was done by the target/reference ratio of calibrator. Here, target/reference value of control sample was used as calibrator. The miRNA snoRNA142 was used as endogenous control. We calculated the fold change using the formula 2^−∆∆Ct^ ^26^. A list of primers used in the study is provided in Supplementary Table 1.

### Target prediction and pathway analysis: Identification of miRNAs involved in CD4+ T cells differentiation and cellular immune responses

For analysis of miRNAs role in cellular immune response and Th1/2 differentiation, we retrieved the signaling pathway of Th1/2 differentiation deposited in KEGG database^27^. We have predicted the targets of dysregulated miRNAs using the online software Target Scan 7.2 and identified transcription factors and genes involved in regulation and differentiation of naive CD4+ T cells into Th1/2 phenotype during *Leishmania donovani* infection. We predicted the regulatory network of identified differentially expressed miRNAs, associated with T cells biology and cellular immune responses.

#### Gene ontology analysis

The gene ontology (GO) and pathway enrichment analysis were carried out for the differentially expressed miRNAs (with statistical significance) using the miRNet^28^. GO analysis was done on the basis of biological process, cellular component and molecular function. KEGG pathway was used for the pathway enrichment analysis. GO and pathway enrichment (with statistical significance, p < 0.05) involved in CD4+ T cell biology was considered for computing the graph.

### Statistical analysis

All the experiments (infection experiments, next-generation sequencing and qPCR) were performed in triplicate and data are represented as mean of three independent experiment. Statistical analysis was performed using Graph Pad Prism 5.0. The significance between means of two groups was analyzed by paired t-test. A p < 0.01 was considered as highly significant, p < 0.05 was considered as significant. The enrichment analysis was based on the hypergeometric test after adjustment for false discovery rate (FDR). Enrichments with the P value < 0.05 was only considered.

## Results

### Confirmation of *L. donovani* infection in macrophages

*Leishmania* infection in macrophages (Mφ) was confirmed by Geimsa staining (Supplementary Fig. 1A–C). In infected Mφ, amastigotes were clearly observed under a bright field microscope.

### Macrophage- CD4+ T cells co-culture

After isolation and purification, the CD4+ T cell population was analyzed by flow cytometry. Approximately 97% cells were found to be CD4+ T positive (Supplementary Fig. 2). Viability was also analyzed and 94% cells were found viable. After co-culture of CD4+ T cells and with *L. donovani* infected and control macrophages, CD4+ T cells were collected (93% purity) and was RNA was isolated for next generation sequencing of miRNAs.

### Next generation sequencing of miRNA

**Figure 1:**
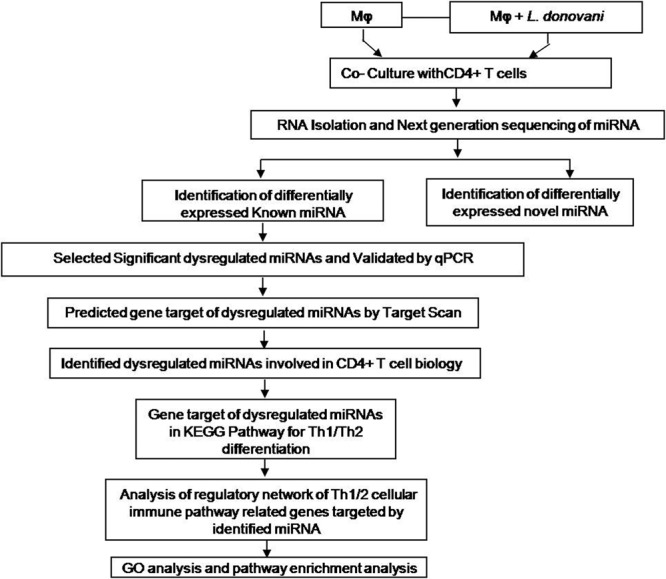
Over view of our experiments: CD4+ T cells were co-cultured with *L. donovani* infected and uninfected macrophages. After 36 hrs of co-culture, both groups of CD4+ T cells were collected for RNA isolation and next generation sequencing of miRNA. The statistically significant (p < 0.05) dysregulated miRNAs were taken for identification of miRNAs involved in CD4+ T cell biology.

The overviews of our experiments are depicted in Fig. 1. Total RNA was isolated from both groups and subjected to next-generation sequencing for the identification of known and novel miRNAs. Total 492406 and 635618 high quality, non-redundant clean reads were generated in control and infected groups for miRNA analysis (Table 1).

**Table 1:** Basic statistics of the reads obtained from NGS.

	Total Reads	Total Unique Reads
Control (CD4+ T cells co-culturedwith macrophages)	13838871	492406
Infected (CD4+ T cells co-cultured with *L. donovani* infected macrophages)	16630829	635618

### Analysis of conserved (Known) miRNA

**Figure 2:**
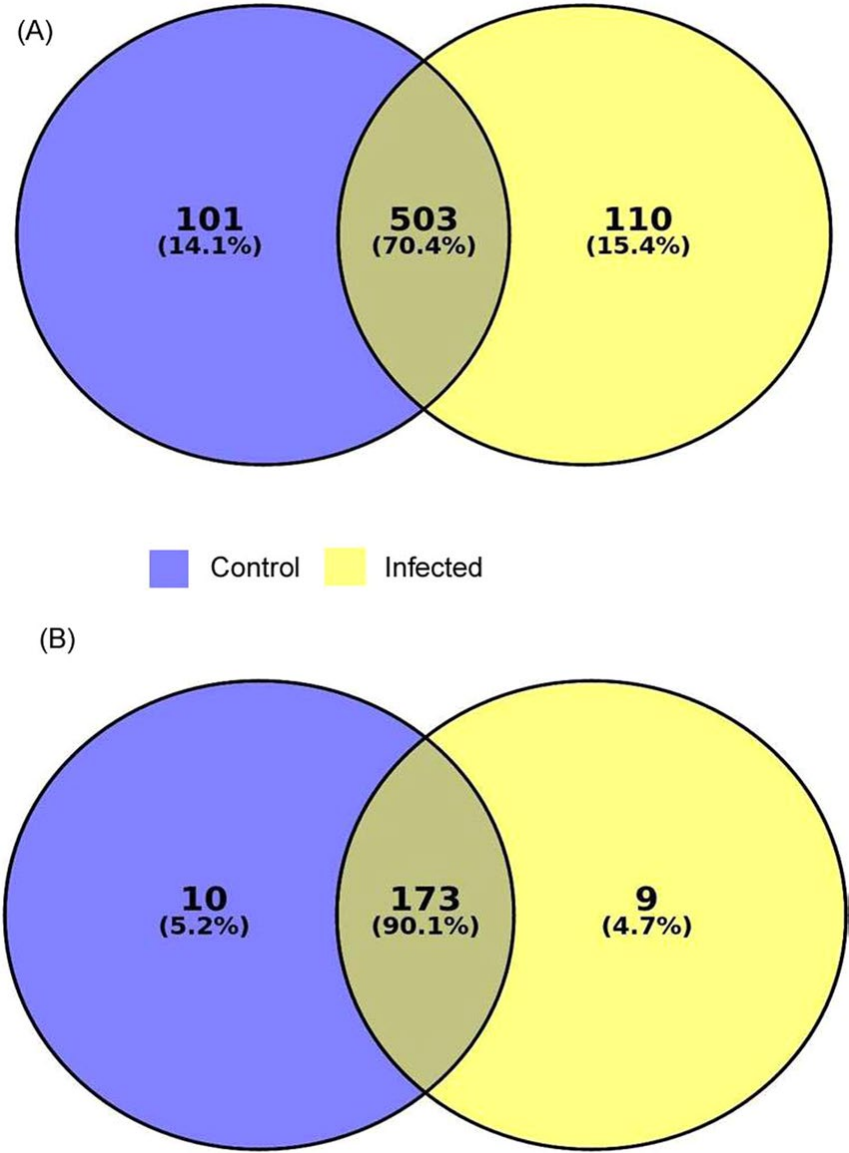
Analysis of next generation sequencing results for known miRNAs. **(A)** Total 604 and 613 miRNA were found in control and infected sample. However, 101 and 110 known miRNAs were expressed only in control and infected sample respectively, 503 miRNAs were common between these two samples. **(B)** Micro RNA Family analysis of known miRNA. The miRNA expressed only in control and infected sample belong to 10 and 9 families respectively. The miRNAs, which were common between two samples, belong to 173 families.

After obtaining of high-quality reads of both groups, the data was analyzed for identification of miRNAs using software BOWTIE 1.1.2 and online available miRbase as the parameter mentioned in the material and method section. A total of 604 and 613 conserved miRNAs were identified in the control and infected sample belonging to 192 families, **(**Fig. 2, Table 2). The 503 miRNAs across 173 families were found common between two samples. Exclusively, 101 and 110 miRNA were found only in control and infected samples respectively. The family analysis revealed that the let-7 family of miRNA was the most abundant family followed by miR-10 and miR-467 **(**Supplementary Fig. 3). The complete list of up and down-regulated conserved miRNAs are mentioned in Supplementary excel sheet 1.

**Table 2:** Raw Read counts (RC) statistics of known and novel miRNA.

	Total miRNAs	miRNAs RC >= 50	miRNAs RC >= 10
**Known miRNA**			
Control (CD4+ T cells co-cultured with macrophages)	604	224	378
Infected (CD4+ T cells co-cultured with *L.donovani* infected macrophages)	613	182	309
**Novel miRNA**			
Control (CD4+ T cells co-cultured with macrophages)	660	12	110
Infected (CD4+ T cells co-cultured with *L.donovani* infected macrophages)	517	10	58

### Novel miRNA prediction

The sequences not found in the miRbase were extracted and were considered for novel miRNA prediction using the BOWTIE, Mireap_0.22b and RNA fold software as mentioned in the material and method section. A total of 660 and 517 novel miRNAs were predicted in control and infected samples respectively (Table 2). The complete list of up and down-regulated novel miRNAs are given in Supplementary Excel Sheet 2.

### Identification of differentially expressed CD4+ T miRNA upon *Leishmania donovani* infection

Here, we report a complete miRNAs expression profile of CD4+ T cells co-cultured with *L. donovani* infected and uninfected macrophages. A total of 1217 known and 1177 novel miRNAs across two groups were identified. Expression analysis identified 112 known and 7 novel up-regulated miRNAs and 96 known and 9 novel down-regulated miRNAs in the infected samples compared to controls. The heat map of conserved and novel miRNAs is shown in Supplementary Figs. 4 and 5 respectively. After filtering the miRNAs for statistical significance (p < 0.05), we obtained a total 19 and 17 up and down-regulated known miRNAs, respectively. A list of statistically significant dysregulated known miRNAs are given in Table 3.

**Table 3:** List of statistically significant (p < 0.05) dysregulated miRNAs by NGS. The dysregulated miRNAs were validated by qPCR, same pattern of miRNAs expression was observed.

	List of Up-regulated miRNA	Fold Change		Log_2_fold change		p-value	
		NGS	qPCR	NGS	qPCR	NGS	qPCR
Up-regulated	mmu-let-7e-3p	2.71	3.32	1.43	1.73	0.035	0.024
	mmu-miR-10a-3p	2.45	3.14	1.29	1.65	0.021	0.032
	mmu-miR-134-5p	6.90	5.98	2.78	2.58	0.025	0.033
	mmu-miR-193a-3p	3.16	3.21	1.66	1.68	0.024	0.019
	mmu-miR-212-3p	2.17	2.42	1.12	1.27	0.046	0.023
	mmu-miR-296-5p	4.21	3.86	2.07	1.94	0.028	0.014
	mmu-miR-33-5p	6.85	6.12	2.77	2.61	0.014	0.028
	mmu-miR-431-5p	2.04	2.22	1.03	1.15	0.025	0.016
	mmu-miR-5128	2.10	2.44	1.07	1.28	0.048	0.016
	mmu-miR-5620-3p	5.51	6.08	2.46	2.60	0.015	0.015
	mmu-miR-574-5p	2.21	2.54	1.07	1.34	0.028	0.015
	mmu-miR-690	2.38	2.12	1.25	1.08	0.032	0.044
	mmu-miR-6994-5p	4.21	3.78	2.07	1.91	0.046	0.035
	mmu-miR-7093-3p	5.26	5.42	2.39	2.43	0.013	0.034
	mmu-miR-7235-3p	2.03	2.16	1.02	1.11	0.024	0.045
	mmu-miR-7673-5p	2.10	2.48	1.07	1.31	0.047	0.018
	mmu-miR-7a-1-3p	2.85	2.62	1.51	1.38	0.043	0.014
	mmu-miR-8094	6.32	5.92	2.66	2.56	0.015	0.036
	mmu-miR-8096	6.32	6.68	2.66	2.73	0.012	0.034
Down-regulated	mmu-let-7j	0.12	0.16	−3.01	−2.64	0.025	0.016
	mmu-miR-145a-5p	0.49	0.38	−1.01	−1.39	0.047	0.011
	mmu-miR-147-3p	0.41	0.48	−1.26	−1.05	0.037	0.041
	mmu-miR-181a-2-3p	0.43	0.22	−1.21	−2.18	0.012	0.048
	mmu-miR-18a-3p	0.40	0.32	−1.29	−1.64	0.049	0.028
	mmu-miR-23b-3p	0.21	0.28	−2.19	−1.83	0.049	0.028
	mmu-miR-322-5p	0.45	0.36	−1.13	−1.47	0.028	0.016
	mmu-miR-340-5p	0.16	0.24	−2.59	−2.05	0.023	0.046
	mmu-miR-3473f	0.38	0.46	−1.38	−1.12	0.016	0.024
	mmu-miR-365-3p	0.49	0.40	−1.01	−1.32	0.026	0.034
	mmu-miR-486a-3p	0.35	0.34	−1.50	−1.55	0.043	0.031
	mmu-miR-503-5p	0.33	0.31	−1.58	−1.68	0.013	0.015
	mmu-miR-615-3p	0.46	0.41	−1.09	−1.28	0.033	0.049
	mmu-miR-7017-5p	0.30	0.39	−1.73	−1.35	0.002	0.016
	mmu-miR-7655-3p	0.35	0.37	−1.50	−1.43	0.004	0.015
	mmu-miR-8115	0.39	0.45	−1.35	−1.15	0.001	0.022
	mmu-miR-93-3p	0.48	0.43	−1.03	−1.21	0.036	0.041

### Real time validation of dysregulated miRNA

**Figure 3:**
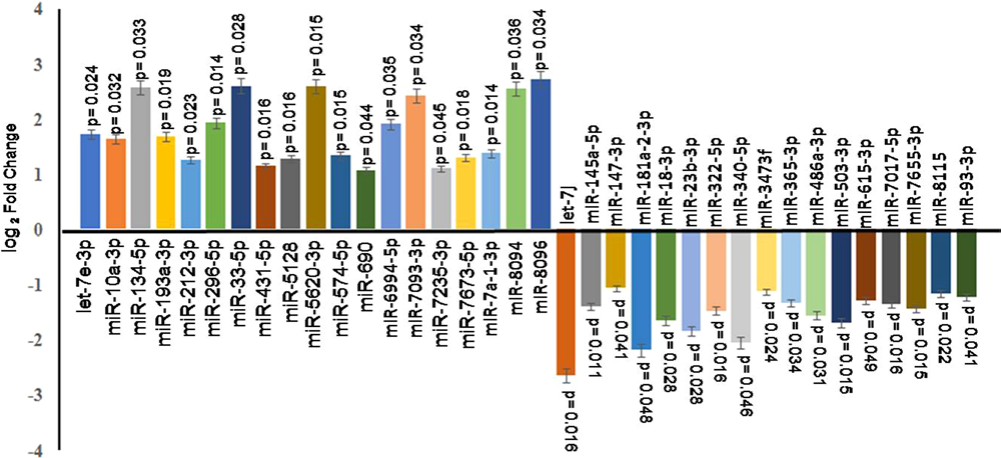
Real time validation of dysregulated miRNAs. CD4+ T cells were co-cultured with *L. donovani* infected and uninfected macrophages. After 36 hrs, CD4+ T cells were taken for RNA isolation and next generation sequencing. In NGS result, we found only 19 up-regulated miRNAs and 17 down-regulated miRNAs were statistically significant (p < 0.05), which were further validated by qPCR. The qPCR showed same expression pattern as we found in NGS. The differential expression analysis of miRNAs are depicted as “log2 fold change”.

In order to validate the significantly dysregulated miRNA, the quantitative real time PCR (qPCR) was performed. The sno RNA142 was taken as endogenous control. The up-regulated miRNAs with log_2_ fold change are; let-7e-3p (1.73 ± 0.21), miR-10a-3p (1.65 ± 0.19), miR-134-5p (2.58 ± 0.29), miR-193a-3p (1.68 ± 0.22), miR-212-3p (1.27 ± 0.19), miR-296-5p (1.94 ± 0.28), miR-33-5p (2.61 ± 0.32), miR-431-5p (1.15 ± 0.19), miR-5128 (1.28 ± 0.21), miR-5620-3p (2.60 ± 0.34), miR-574-5p (1.34 ± 0.22), miR-690 (1.08 ± 0.16), miR-6994-5p (1.91 ± 0.28), miR-7093-3p (2.43 ± 0.36), miR-7235-5p (1.11 ± 0.21), miR-7673-5p (1.31 ± 0.22), miR-7a-1-3p (1.38 ± 0.24), miR-8094 (2.56 ± 0.28) and miR-8096 (2.73 ± 0.32). The down-regulated miRNA with log_2_ fold change are; mmu-let-7j (−2.64 ± 0.32), mmu-miR-145a-5p (−1.39 ± 0.18), miR-147-3p (−1.05 ± 0.14), miR-181a-2-3p (−2.18 ± 0.28), miR-18a-3p (−1.64 ± 0.22), miR-23b-3p (−1.83 ± 0.26), miR-322-5p (−1.47 ± 0.19), mmu-miR-340-5p (−2.05 ± 0.28), miR-3473f (−1.12 ± 0.19), miR-365-3p (−1.32 ± 0.22), miR-486a-3p (−1.55 ± 0.21), miR-503-5p (−1.68 ± 0.19), miR-615-3p (−1.28 ± 0.18), miR-7017-5p (−1.35 ± 0.21), miR-7655-3p (−1.43 ± 0.22), miR-8115 (−1.15 ± 0.18) and miR-93-3p (−1.21 ± 0.18) (Fig. 3**)**.

### The miRNAs were involved in Th1 and Th2 differentiation during *Leishmania* infection

We predicted the targets of dysregulated miRNAs using online Target Scan software and found that 11 up-regulated and 9 down- regulated miRNAs which target transcription factors responsible for CD4 T cell differentiation as mentioned in the KEGG pathway.

**Figure 4:**
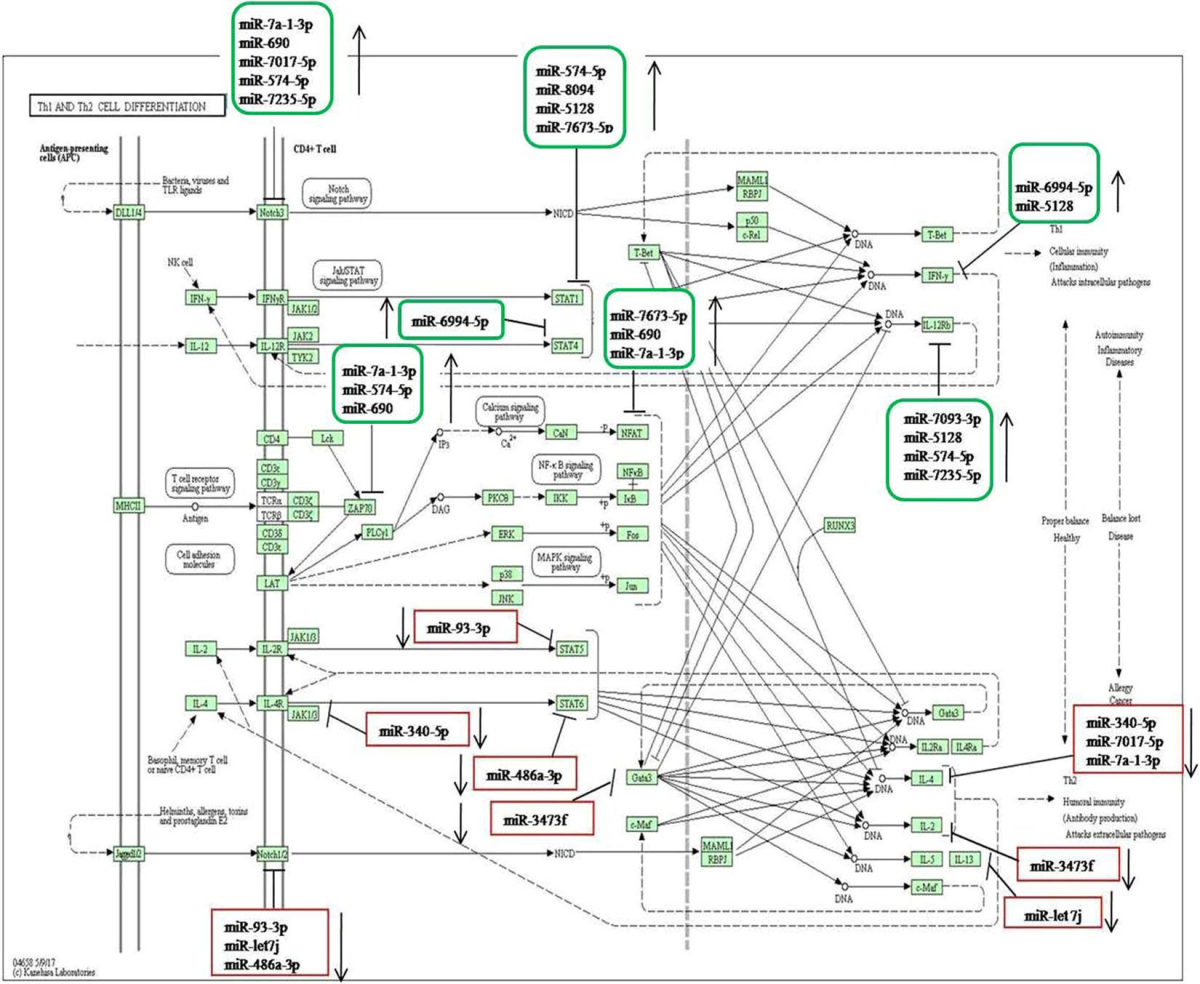
The KEGG pathway (Th1and Th2 cell differentiation-Mus musculus, 04658) retrieved from KEGG data base of Kanehisa Laboratories^27^. We predicted the gene target (transcription factors involved in Th1and Th2 cell differentiation) of dysregulated miRNAs by Target Scan 7.2 software. The up regulated miRNAs shown in green box, targets transcription factors which promote naive CD4+ T cell to Th1 phenotype. The down regulated miRNA shown in red box, targets transcription factors which promote naïve CD4+ T cell to Th2 population.

The up-regulated miRNAs target transcription factors (STAT 1, STAT 4, Notch 3, IL-12rb, ZAP 70 and IFN-γ) which directly or indirectly are involved in transformation of CD4+ T cells to Th1 phenotype while down-regulated miRNA paired with transcription factors (STAT 5, STAT 6, GATA 3, Notch ½, IL-2, IL-4, IL-13 and Jak1/3) those are involved in differentiation of CD4+ T cells into Th2 population **(**Fig. 4**)**. These results suggest that miRNAs regulate transcription factors that direct CD4+ T cells towards Th1 or Th2 phenotypes albeit more detailed investigation are needed to confirm these results.

**Figure 5:**
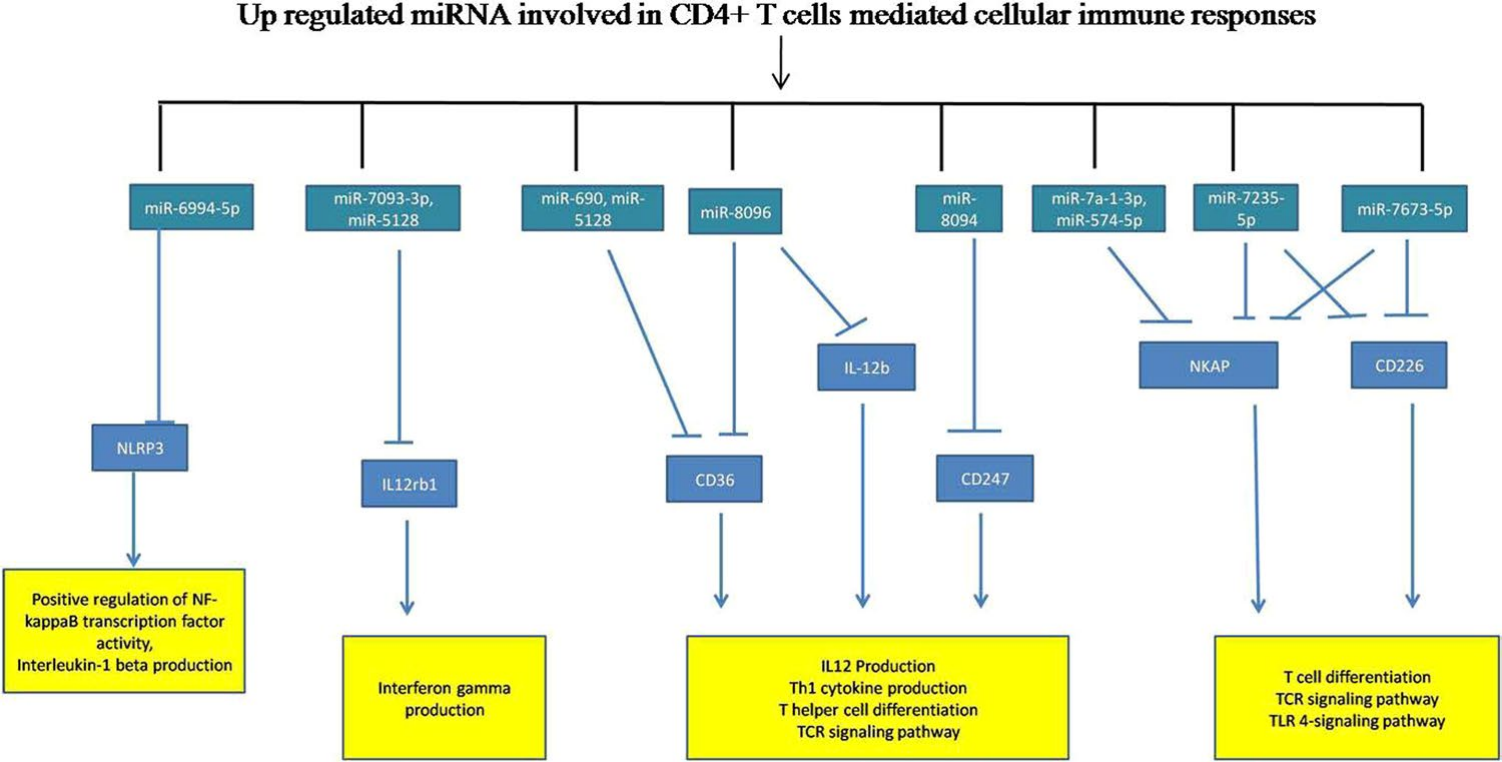
miRNA-regulatory network regulated by up-regulated miRNAs of CD4+ T cells. The target prediction of up-regulated miRNAs by Target Scan 7.2 software. We analyzed the immunological role of the genes targeted by up-regulated miRNAs filtered out on the basis of function linked to cellular immune responses and signalling pathways involved in *Leishmania donovani* infection.

**Figure 6:**
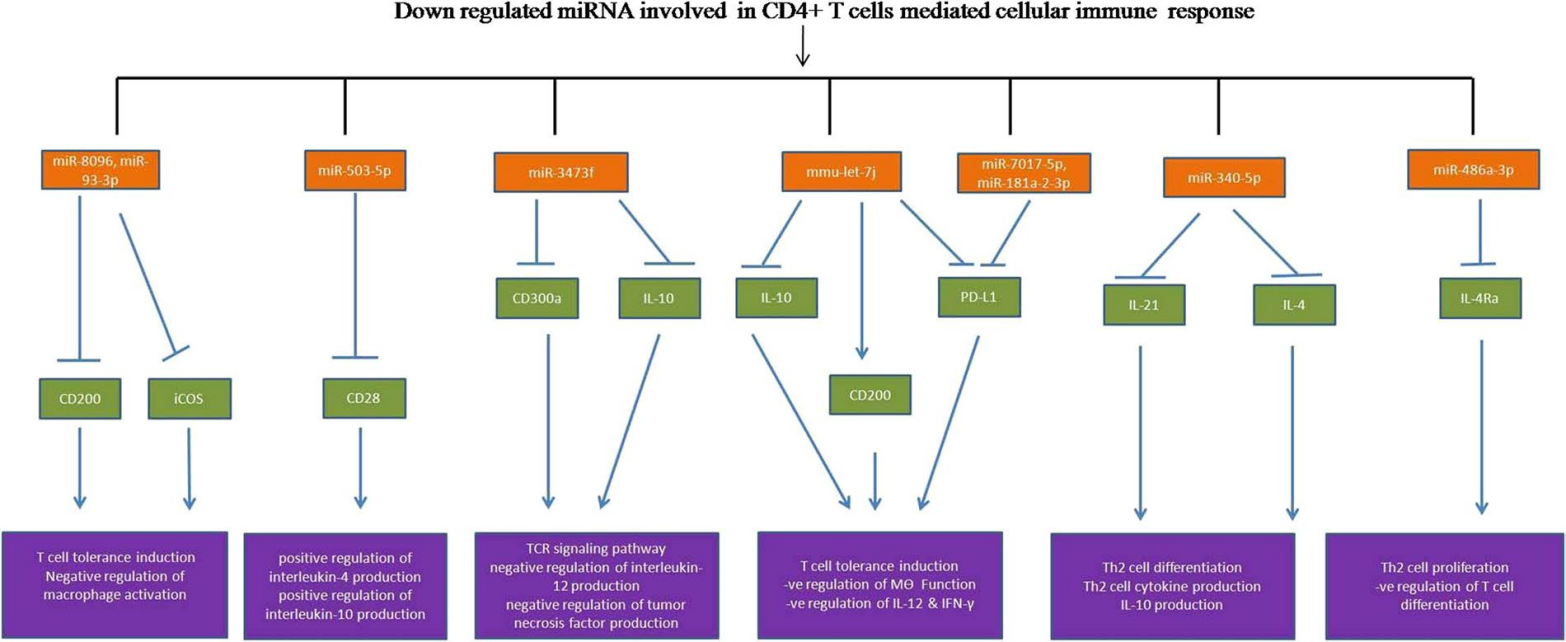
miRNA-regulatory network regulated by down-regulated miRNAs. The target prediction of down-regulated miRNA by online software Target Scan 7.2. We analyzed the immunological role of the gene targeted by down-regulated miRNAs and filtered out on the basis of function linked to cellular immune responses and signalling pathways involved in *Leishmania donovani* infection.

The identified up and down-regulated miRNA have targets that are directly linked to T cell differentiation, IFN-γ signaling, TCR signaling, and tolerance induction pathway. The regulatory network and their diagrammatic representation of up and down-regulated miRNAs are shown in Figs. 5 and 6.

### MicroRNA target prediction and their validation

**Figure 7:**
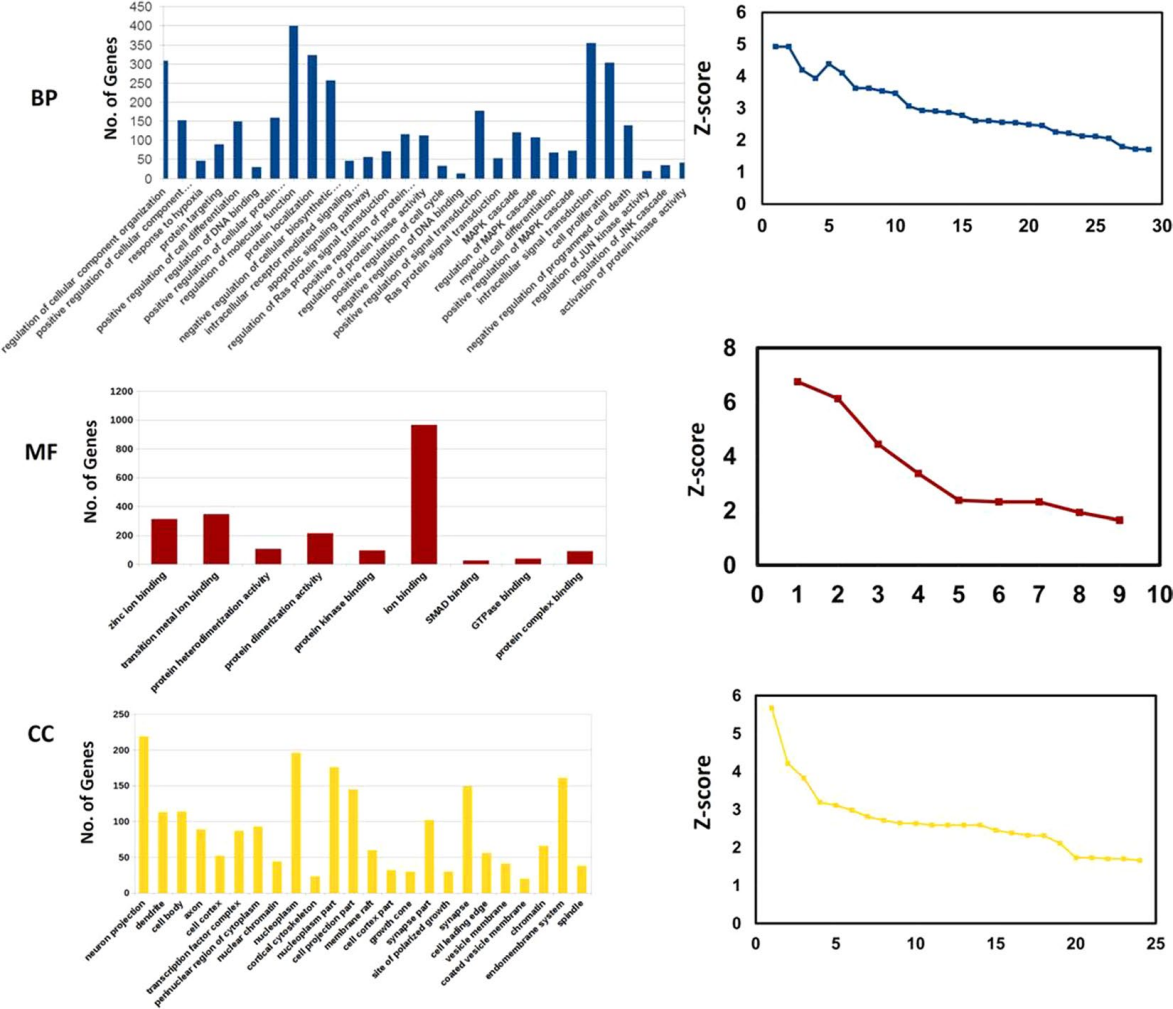
GO enrichment analysis of dysregulated miRNA-target genes involved in CD4+ T cells biology. The target genes were categorized on the basis of Biological Process (BP), Molecular Function (MF) and Cellular components (CC). The statistically significant (p < 0.05) genes of Biological Process, Molecular Function and Cellular components were taken for computing the graph.

**Figure 8:**
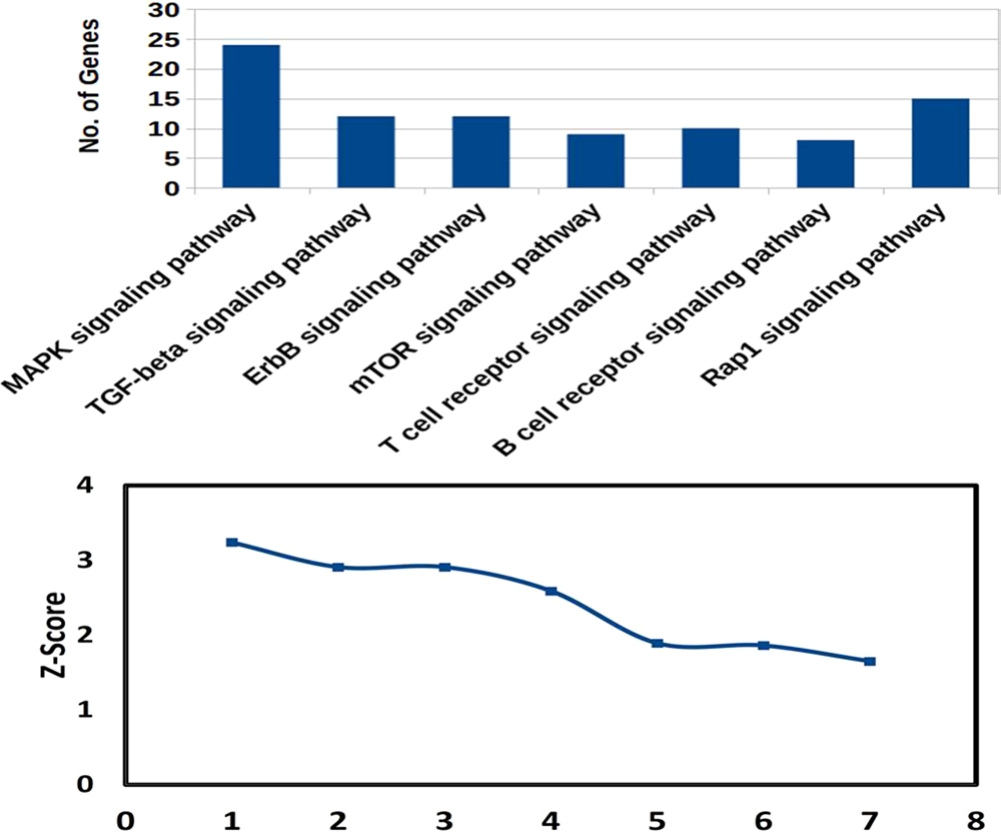
Pathway enrichment analysis of dysregulated miRNAs. We predicted the pathways involved in CD4+ T cells biology and significant pathway (p < 0.05) were taken for computing the graph. Important pathways like MAPK signaling pathway, T cell receptor pathway, mTOR signalling pathway and other important pathways were targeted by dysregulated miRNAs.

We have listed the identified target genes of up and down-regulated miRNAs and further validated them using the module of miRBase database and miR-mRNA extract. We have identified several significant transcripts that were targeted by identified miRNA. The mRNA-miRNA interaction was categorized on the basis of their molecular functions, biological processes and cellular components. After GO and pathway enrichment, we selected only statistical significant (p < 0.05) gene and pathways involved in CD4+ T cell mediated cellular immune responses (Figs. 7 and 8). The biological process, molecular function, and cellular component of CD+4 T cells and targeted by differentially expressed miRNAs are listed in Supplementary Excels Sheets 3 and the list of significant pathway involved in CD4+ T cell biology are listed in Supplementary Excels Sheets 4. After GO enrichment, we identified significantlly differentially expressed miRNAs on the basis of their various biological processes linked to CD4+ T cells. The differentially expressed miRNAs regulate apoptotic signaling, cell proliferation, activation of protein kinase activity, regulation of JUN kinase activity and MAPK pathway and other biological processes. These all biological processes are important for either parasite clearing or pathogenesis of the disease.

The molecular functions linked with differentially expressed miRNAs are protein kinase activity, protein dimerization activity, protein heterodimerization activity, Smad binding and other molecular functions. These all the molecular functions are vital to restrict intracellular parasites survival. Hence, it is of great significance to decipher how these altered miRNAs regulate CD4+ T-cell-mediated cellular immune responses. The differentially expressed miRNAs also target genes located in the various cellular components.

## Discussion

In the last two decades after the discovery of miRNA, significant research has been done in exploring their role in human diseases especially in cancer^29^. However, only a few studies are available in case of parasitic diseases like leishmaniasis^30,31^. CD4+ T cells represent one of the main targets of *Leishmania* parasites, which co-opts the process of cell activation and differentiation for the establishment and progress of the disease^32^. To identify the microRNAs that are potentially linked to T-cell activation and *Leishmania* replication, we profiled miRNA expression in purified CD4+ T cells using high-depth small RNA-sequencing and in-silico approach. Here, we provide the first comprehensive data of the miRNA expression in purified mice CD4+ T cells in the case of *Leishmania donovani* infection.

We have identified a total of 604 and 613 conserved miRNAs in control and infected samples, respectively belonging to 192 miRNA families in CD4+ T cells during *Leishmania* infection using NGS. We have also identified their target genes involved in cellular immune response and Th1 and Th2 differentiation. Compared with traditional miRNA array technique, the advantage of NGS is in identification of novel miRNAs, which have not been annotated in miRNAs databases. The current form of NGS combined with the use of bioinformatics tools enables deep investigation and a clear understanding of the gene regulation by miRNAs and discriminates the closely related small RNAs and for detection of novel small RNAs^33,34^. Our results showed that enormous quantities of miRNAs, mostly of let 7 families, were differentially expressed. The members of this family of miRNAs are actively engaged in several signaling pathways such as MAPK and NF-kB which regulates pro-inflammatory immune responses^35^.

The major cells that participate in *Leishmania* pathogenesis and clearance of parasites are T cells, macrophages and NK cells^36^. The CD4+ T cells play a key role in *Leishmania* infection and secrete several cytokines, which are deciding factors in parasite clearance or pathogenesis of the disease^37^. In this study, out of the total identified differentially expressed miRNA, 19 up-regulated and 17 down-regulated miRNAs were found significant, however, only 11 up-regulated and 9 down-regulated miRNAs targeted genes of CD4+ T differentiation.

The differentiation of naive CD4+ T cells into Th1/2 cytokines is tightly regulated; however, miRNA mediated regulations are yet not understood in *Leishmania donovani* infection^38^. Differentiation of naive CD4+ T cells to the Th1/2 phenotype involves notch proteins associated pathway, JAK-STAT pathway, and MAPK signaling pathway^39^. The transcription factors involved in these pathways are notch 3, STAT1, STAT4, JAK1/2, ZAP70, which help the differentiation of CD4+ T cells to Th1 cytokines whereas differentiation of Th2 phenotypes occurs with the help of transcription factors Notch 1/2, STAT 5, STAT6, GATA3^39^. In this study, the up-regulated miRNAs (miR-7a-1-3p, miR-690, miR-7017-5p, miR-574-5p and miR-7235-5p) target notch 3 gene. Notch 3 has a major role in Th1 immune response such as IFN-γ production. However, in *Leishmania* infection, miRNA suppresses the notch 3 gene and impair the production of IFN-γ thus creating favorable conditions for the survival of the parasites. STAT 4 and STAT1 has a major role in JAK-STAT signaling for IFN-γ production. Our study suggests that these genes are controlled by miR-574 and miR-6994-5p.

IFN-γ is the main pro-inflammatory cytokine secreted by CD4+ T cells, which helps in clearing *Leishmania* parasites during infection. In this study, we found that miRNA-6994-5p and miR-5128 target IFN-γ gene. Moreover, we observed that the up-regulated miRNAs (miR-7093-3p, miR-5128, miR-574-5p and miR-7235) target interleukin-12 receptor and may therefore, deregulate the IFN-γ mediated signaling. The upregulated miRNA also targets the genes like nuclear factor of activated T cells (NFAT) and Zeta chain associated protein kinase 70 (ZAP 70) which mediates also IFN-γ and T cell-mediated signaling. Overall, our results showed that the up-regulated miRNAs target genes linked to IFN-γ pathway, therefore further studies on their regulatory role in IFN-γ production is needed.

Further, we observed that the differentiation of naive CD4+ T cells into the Th2 phenotype was controlled by down-regulated miRNAs. The miR-340-5p targets the IL-4, a major Th2 cytokines secreted by CD4+ T cells. It is well known that in *Leishmania* infection, Th1 cytokines polarized to Th1 cytokines that favor parasite survival. Here, miR-340-5p promotes the IL-4 production by its down-regulating its function.

The IL-2 and IL-13 are major Th2 cytokines secreted by CD4+ T cells^40,41^, however, they are targeted by down-regulated miRNA (miR-3473f and let 7j). Our results indicated that down-regulated miRNAs regulate differentiation of naive CD4+ T cell to Th2 phenotype. Therefore, further investigation is needed for understanding their role in *Leishmania* induced Th2 immune responses. MiRNA-93-3p and 486a-3p targets STAT 5 and STAT 6 genes, which are important transcription factors for Th2 differentiation. Since these two miRNAs were down-regulated in infected CD4+ T cells, we conclude that there may be a possible role of these miRNAs in the regulation of T cell proliferation, differentiation and Th1/Th2 dichotomy in *Leishmania* pathogenesis. Notch ½ and GATA 3 are important genes that participate in the differentiation of Th2 phenotype from CD4+ T cells^42^. In this study, we found that down-regulated miRNAs (miR-93-3p, let 7j, 486a-3p and miR-3473f) target the above said transcription factors. Our results indicate that in the case of *Leishmania* infection, down-regulation of these miRNA leads to the differentiation of CD4+ T cells into the Th2 phenotype.

We also found that the significantly dysregulated miRNAs involved in CD4+ T cells biology target NLRP3, ILrb1, CD36, IL-12rb, CD247, NKAP, CD200, iCOS, CD28, CD300a, IL-10, PDL1, IL-21, IL-4 and IL-4ra genes. These genes regulate the CD+4 T cell differentiation and coordinate the immunological pathway with other cells^43–46^. The current results suggest that in *Leishmania* infection, several immunological pathways gets activated which help either in disease establishment or clearance of the parasite and regulated through these small non-coding small RNAs.

The targeted genes of up and down-regulated miRNAs were further classified on the basis of their function in T cells biology viz. molecular function, biological processes and cellular components. Using Insilco analysis revealed that the differentially expressed miRNAs play a crucial role in the regulation of protein kinas-C activity, cell proliferation, regulation of MAPK kinase cascade, Smad binding, protein dimerization activity and cellular component of CD4+ T cell biology. Our results thus suggest that miRNAs may regulate several cascades (molecular function, biological processes and cellular components) of T cells biology, which are important for immunological balance in *Leishmania* infection.

Together, our findings suggest that miRNAs are the master regulator of CD4+ T mediated immune response in visceral leishmaniasis. We found that 11 up-regulated and 9 down-regulated miRNAs are involved in CD4+ T cells biology. The identified up-regulated miRNAs probably suppress transcription factors that are involved in the differentiation of naive CD4+ T cells into Th1 phenotype whereas down-regulated miRNAs target transcription factors involved in the transformation of naive CD+ T cells to Th2 phenotype. These results indicate that during the course of *Leishmania* infection, miRNAs may strongly regulate differentiation of naive CD4+ T to Th1/Th2 phenotype and immune response. Further, detailed studies are needed to clearly demonstrate the function of these miRNAs in the regulation of different transcription factors leading to the development of visceral leishmaniasis.

## Supplementary information

Supplementary Information.

Supplementary excel sheet 1.

Supplementary excel sheet 2.

Supplementary excel sheet 3.

Supplementary Excel Sheet 4.
