# Supplementary material for: Leishmania donovani infection induce differential miRNA expression in CD4+ T cells
Source: Sci Rep. 2020 Feb 26;10:3523. doi: 10.1038/s41598-020-60435-2 (PMC7044172; doi:10.1038/s41598-020-60435-2)
Supplement: Supplementary file 1 — Supplementary Information. [file 41598_2020_60435_MOESM1_ESM.pdf]

Leishmania donovani infection induce differential miRNA expression in CD4+ T cells.

Vinod Kumar[1], Sushmita Das[2], Ajay Kumar[1], Neeraj Tiwari[5], Ashish Kumar[3], Kumar Abhishek[1], Abhishek Mandal[1], Manjay Kumar[1], Taj Shafi[1], Tanvir Bamra[1], Rakesh Kumar Singh[5], Vijayakumar Sarvanan[4], Abhik Sen[1], Pradeep Das[1]*

[1]Division of Molecular Biology, Rajendra Memorial Research Institute of Medical Sciences, Agamkuan, Patna, Bihar, India

[2]Department of Microbiology, All India Institute of Medical Sciences, Phulwarisharif, Patna Bihar, India

[3]Division of Biochemistry, Rajendra Memorial Research Institute of Medical Sciences, Agamkuan, Patna, Bihar, India

[4]Division of Bioinformatics, Rajendra Memorial Research Institute of Medical Sciences, Agamkuan, Patna, Bihar, India

[5]Department of Biochemistry, Institute of Science, Banaras Hindu University, Varanasi-221005

***Corresponding Author**

Dr. Pradeep Das

Director

Mailing Address: Rajendra Memorial Research Institute of Medical Sciences,

Agamkuan, Patna- 800007, Bihar, India

Telephone No. +91-612-2631565

Fax No. +91-612-2634379

E-mail ID: drpradeep.das@gmail.com

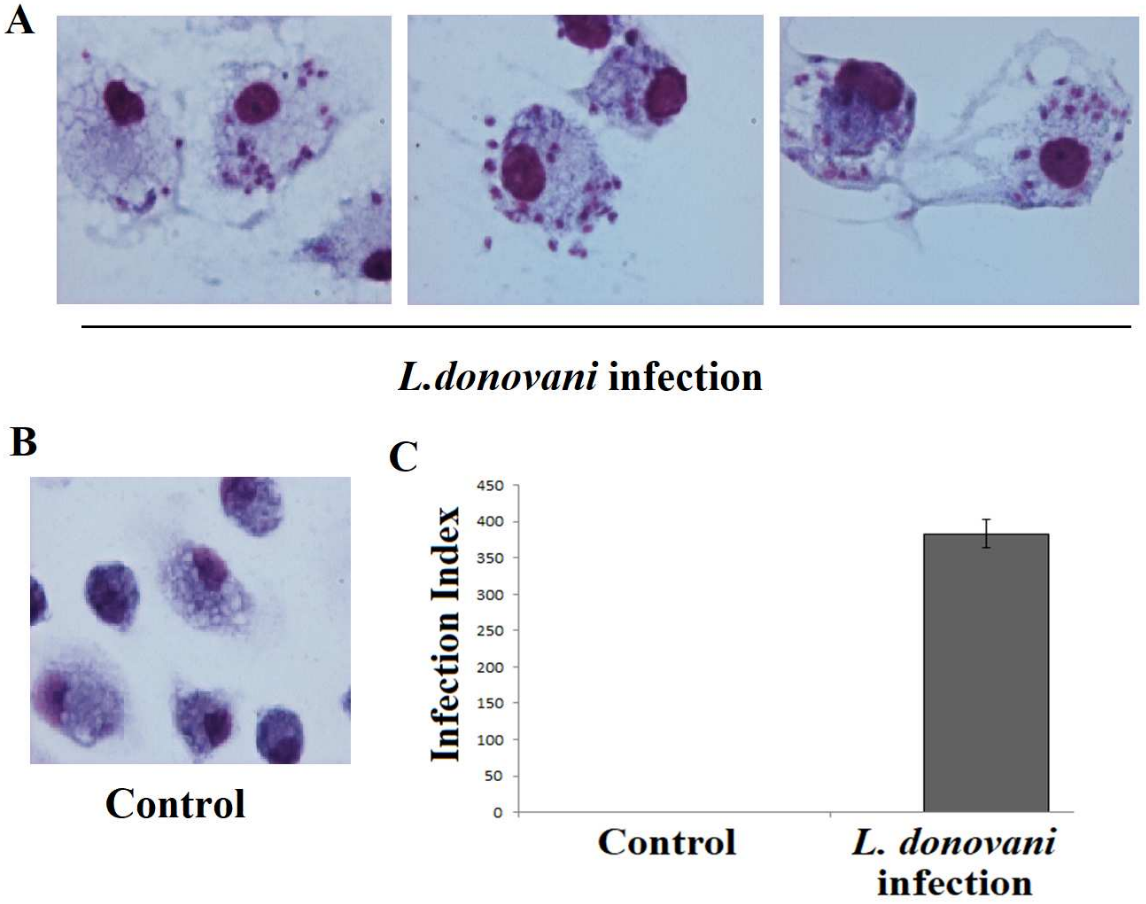

Supplementary Figure 1. Peritoneal macrophages of BALB/c mice were infected with *L. donovani*, uninfected macrophage were taken as control. After 6h of interaction, uninfected parasites were washed and incubated for 24hrs in 5% CO_2 atmosphere. After 24hrs, cells were stained with May-Grunwald, followed by Geimsa staining. (A) The infected macrophages from three different microscopic fields were taken. (B) Control macrophages without infection, (C) Infected cells were counted and results are expressed as infection index. Infection index was measured as mean of infected cells X the mean number of parasites per infected cells. Bar represent mean ± SE of the three independent experiments.

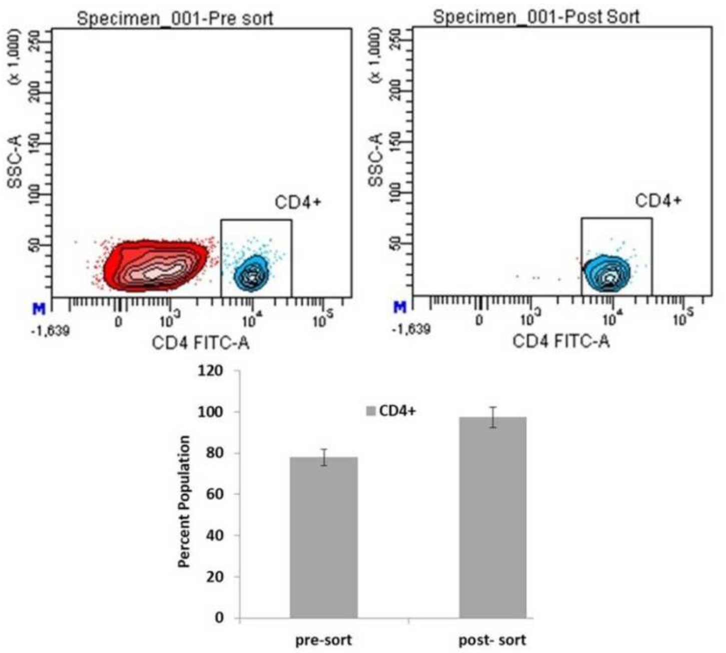

Supplementary Figure 2. (A) Flow cytometry analysis for measurement of CD4+ T cell before and after sorting (B). Graphical representation of CD4+ T cell population.

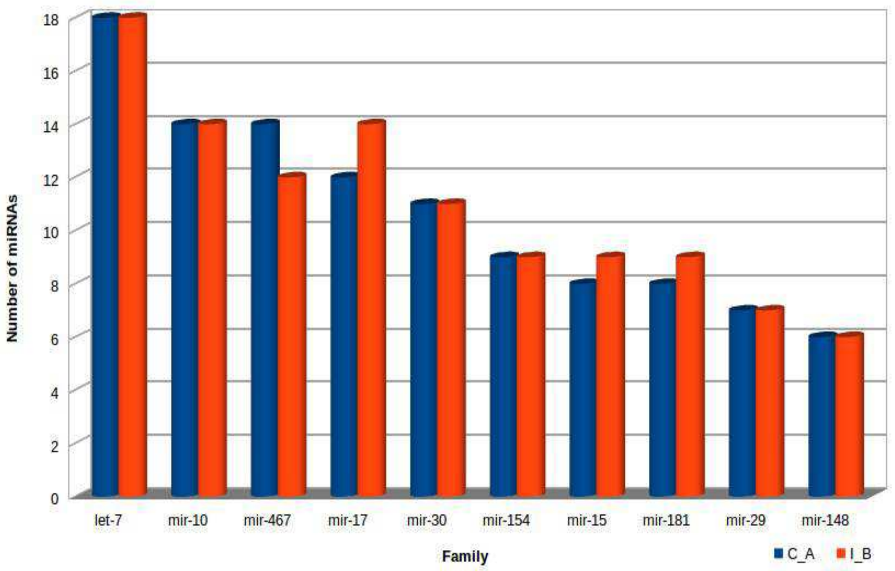

Supplementary Figure 3. Family analysis of conserved miRNA

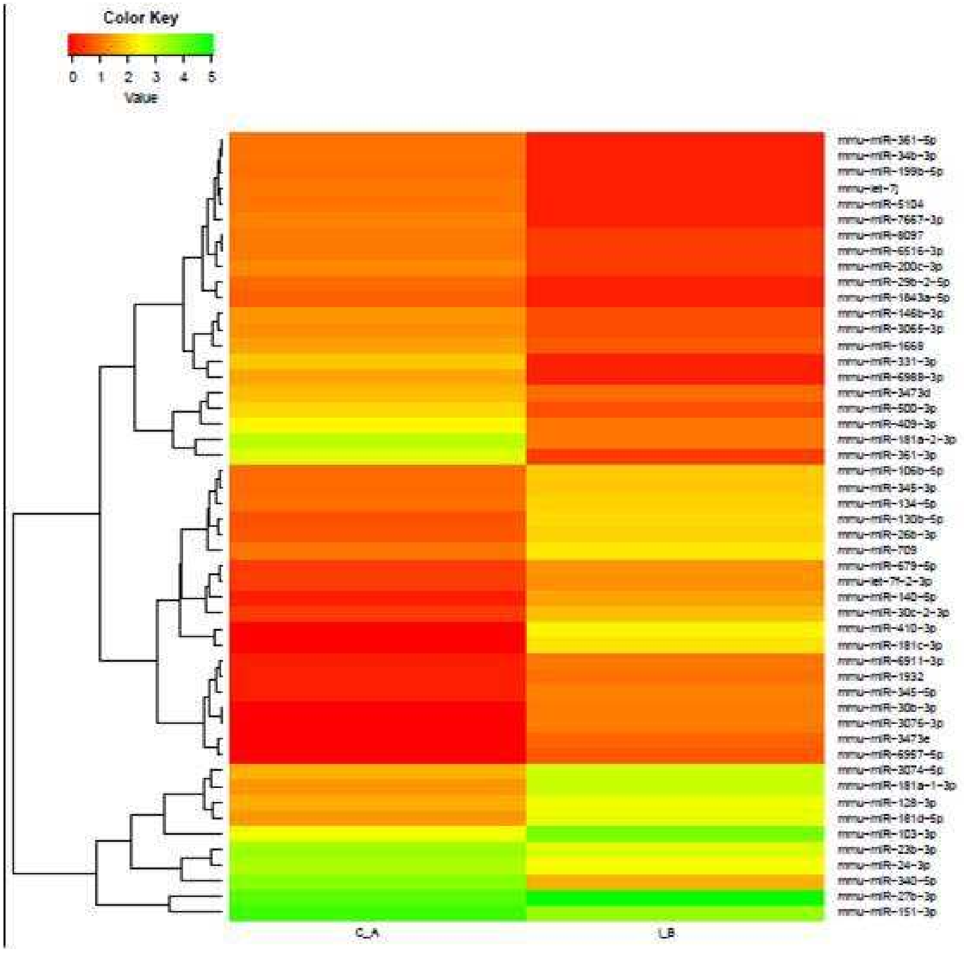

Supplementary Figure 4: Heatmap of known miRNA

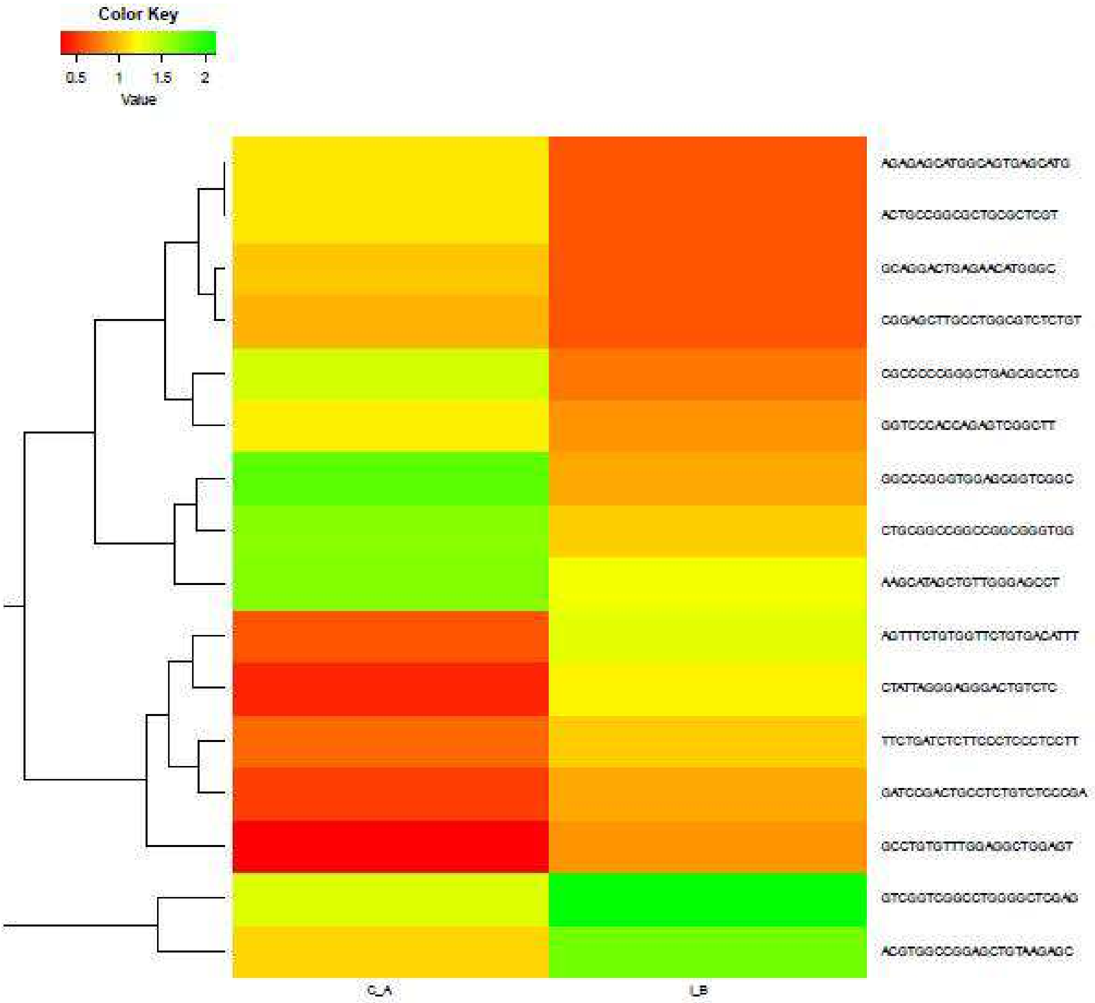

Supplementary Figure 5. Heatmap of novel miRNA

Supplementary Table-1: List of primers used in this study

S. no	List of Up regulated miRNA	Stem Loop Primer Sequence	Forward Primer Seq
1	let-7e-3p	5’GTTGGCTCTGGTGCAGGGTCCGAGGTATTCGCACCAGAGCCAAC GGAAAG 3’	5’ACAAGUCUAUACGGCCUC 3’
2	miR-10a-3p	5’GTTGGCTCTGGTGCAGGGTCCGAGGTATTCGCACCAGAGCCAAC TATTCC 3’	5’CCCGGACAAAUUCGUAUC 3’
3	miR-134-5p	5’GTTGGCTCTGGTGCAGGGTCCGAGGTATTCGCACCAGAGCCAAC CCCCUC 3’	5’CGAUGAUGUGACUGGUUG 3’
4	miR-193a-3p	5’GTTGGCTCTGGTGCAGGGTCCGAGGTATTCGCACCAGAGCCAAC ACUGGG 3’	5’CACUCUAACUGGCCUACA 3’
5	miR-212-3p	5’GTTGGCTCTGGTGCAGGGTCCGAGGTATTCGCACCAGAGCCAAC UGGCCG 3’	5’CCGAUGUAACAGUCUCCA 3’
6	miR-296-5p	5’GTTGGCTCTGGTGCAGGGTCCGAGGTATTCGCACCAGAGCCAAC ACAGGA 3’	5’UAUUUUAGGGCCCCCCCU 3’
7	miR-33-5p	5’GTTGGCTCTGGTGCAGGGTCCGAGGTATTCGCACCAGAGCCAAC UGCAAU 3’	5’CGAAUCGUGCAUUGUAGU 3’
8	miR-431-5p	5’GTTGGCTCTGGTGCAGGGTCCGAGGTATTCGCACCAGAGCCAAC UGCAUG 3’	5’AACUUAUGUCUUGCAGGC 3’
9	miR-5128	5′- GTCGTATCCAGTGCAGGGTCCGAGGTATTCGCACTGGATACGACAGCCAT 3’	5’ GACCCGCAAAAGGGGCAG 3’
10	miR-5620-3p	5′- GTCGTATCCAGTGCAGGGTCCGAGGTATTCGCACTGGATACGACCACAGT 3’	5’ GGAGCTACGAGGCAGGGG 3’
11	miR-574-5p	5′- GTCGTATCCAGTGCAGGGTCCGAGGTATTCGCACTGGATACGACACACAC 3’	5’ GCCGCGTGAGTGTGTGTG 3’
12	miR-690	5′- GTCGTATCCAGTGCAGGGTCCGAGGTATTCGCACTGGATACGACTTTGGT 3’	5’ CCGGCGAAAGGCTAGGCT 3’
13	miR-6994-5p	5′- GTCGTATCCAGTGCAGGGTCCGAGGTATTCGCACTGGATACGACTCACCG 3’	5’ GCGGCAACAAAGGTGGGT 3’
14	miR-7093-3p	5′- GTCGTATCCAGTGCAGGGTCCGAGGTATTCGCACTGGATACGACCTGCAG 3’	5’ GCGCGCTTTCCATCTGTC 3’
15	miR-7235-5p	5′- GTCGTATCCAGTGCAGGGTCCGAGGTATTCGCACTGGATACGACGCCCAG 3’	5’ CCAAACGGAGGGAGGGGT 3’
16	miR-7673-5p	5′- GTCGTATCCAGTGCAGGGTCCGAGGTATTCGCACTGGATACGACCATTCC 3’	5’ GGCCGGTTTGACTGAGAG 3’
17	miR-7a-1-3p	5′- GTCGTATCCAGTGCAGGGTCCGAGGTATTCGCACTGGATACGACTTTGGC 3’	5’ CGCGCGCAACAAAACACA 3’
18	miR-8096	5′- GTCGTATCCAGTGCAGGGTCCGAGGTATTCGCACTGGATACGACTCTCTT 3’	5’ AGACGTGGGCACGGAAGC 3’
19	miR-8094	5′- GTCGTATCCAGTGCAGGGTCCGAGGTATTCGCACTGGATACGACTCTTCT 3’	5’ CGCCGCAACUGAAGGACA 3’
	**List of down regulated miRNA**	**Stem Loop Primer Sequence**	**Forward Primer Seq**
1	let 7j	5′- GTCGTATCCAGTGCAGGGTCCGAGGTATTCGCACTGGATACGACATAACA 3’	5’GGCCCATGAGGTATTAGT 3’

2	miR-145a-5p	5’GTTGGCTCTGGTGCAGGGTCCGAGGTATTCGCACCAG AGCCAAC AGGGAU 3’	5’CAAGCUGUCCAGUUUU CC 3’
3	miR-147-3p	5’GTTGGCTCTGGTGCAGGGTCCGAGGTATTCGCACCAG AGCCAAC UAGCAG 3’	5’AUGCUAGUGUGCGGA AAU 3’
4	miR-181a-2-3p	5′- GTC GTA TCC AGT GCA GGG TCC GAG GTA TTC GCA CTG GAT ACG AC GGTACA 3’	5’AATTTAAACCACCGAC CGT 3’
5	miR-18a-3p	5’GTTGGCTCTGGTGCAGGGTCCGAGGTATTCGCACCAG AGCCAAC CAGAAG 3’	5’CAAUCGACUGCCCUAA GU 3’
6	miR-23b-3p	5’GTTGGCTCTGGTGCAGGGTCCGAGGTATTCGCACCAG AGCCAACGGUAAU 3’	5’CAAUGCAUCACAUUGC CA 3’
7	miR-322-5p	5’GTTGGCTCTGGTGCAGGGTCCGAGGTATTCGCACCAG AGCCAAC UCCAAA 3’	5’CGUAGUCAGCAGCAAU UC 3’
8	miR-340-5p	5′- GTCGTATCCAGTGCAGGGTCCGAGGTATTCGCACTG GAT ACGAC AATCAG 3’	5’GCCCGCTTATAAAGCA AT 3’
9	miR-3473f	5′- GTCGTATCCAGTGCAGGGTCCGAGGTATTCGCACTG GATACGACCATCTC 3’	5’GCCCAACAAATAGGAC TG 3’
10	miR-365-3p	5’GTTGGCTCTGGTGCAGGGTCCGAGGTATTCGCACCAG AGCCAACAUAAGG 3’	5’CGGAUCUAAUGCCCCU AA 3’
11	miR-486a-3p	5′- GTCGTATCCAGTGCAGGGTCCGAGGTATTCGCACTG GATACGACATCCTG 3’	5’ATATATCGGGGCAGCT CA 3’
12	miR-503-3p	5′- GTCGTATCCAGTGCAGGGTCCGAGGTATTCGCACTG GATACGACCCAGGC 3’	5’GGGCGAGAGTATTGTT TC 3’
13	miR-615-3p	5’GTTGGCTCTGGTGCAGGGTCCGAGGTATTCGCACCAG AGCCAACAAGAGG 3’	5’UUAUAAUCCGAGCCUG GG 3’
14	miR-7017-5p	5′- GTCGTATCCAGTGCAGGGTCCGAGGTATTCGCACTG GATACGACACAGCC 3’	5’GCATACAGAGGGTTGT GA 3’
15	miR-7655	5′- GTCGTATCCAGTGCAGGGTCCGAGGTATTCGCACTG GATACGACTCTTTA 3’	5’ TATAAT CGGCCCACGGAG 3’
16	miR-8115	5’GTTGGCTCTGGTGCAGGGTCCGAGGTATTCGCACCAG AGCCAACAGAGCC 3’	5’GUAAGUCCGCAAUAGC AA 3’
17	miR-93-3p	5′- GTCGTATCCAGTGCAGGGTCCGAGGTATTCGCACTG GATACGACCGGGAA 3’	5’CATGGACTGCTGAGCT A 3’
	**Endogenous Control**		
1.	Sno-142	5’GTTGGCTCTGGTGCAGGGTCCGAGGTATTCGCACCAG AGCCAACTTCCTC 3’	5’GCGGCGGGTCAGTGCC ACGTGT 3’
1.		**Universal Reverse Primer Seq.** 5’ GTGCAGGGTCCGAGG 3’	
